# Comparison of superstimulatory protocols with different doses of eCG on ovarian response and oocyte recovery by follicular aspiration in llamas (*Lama glama*)

**DOI:** 10.1590/1984-3143-AR2022-0134

**Published:** 2024-03-08

**Authors:** Uri Harold Perez Guerra, Yesenia María Quispe Barriga, Natalio Luque Mamani, Domingo Alberto Ruelas Calloapaza, Jesús Manuel Palomino Cano, Manuel Guido Pérez Durand

**Affiliations:** 1 Facultad de Medicina Veterinaria y Zootecnia, Universidad Nacional del Altiplano, Puno, Perú; 2 Carrera de Medicina Veterinaria y Zootecnia, Universidad Científica del Sur, Lima, Perú

**Keywords:** eCG dosage, follicle size, ovum pick up, camelids

## Abstract

The objective of this study was to evaluate the effect of three doses of equine chorionic gonadotropin (eCG) for ovarian superstimulation on ovarian response, follicular development and cumulus-oocyte complexes (COCs) collection in llamas. For this purpose, eighteen multiparous non-lactating adult (4–7 yo) female llamas with an average body condition of 2.8 (BCS 1–5) were submitted to a follicular ablation (FA) to induce a new follicular wave emergence. Two days after FA (Day 0), synchronized llamas were randomly allocated to three treatment groups (n = 6/group) and given 500, 750 and 1000 IU of eCG (Novormon®, Syntex, Buenos Aires, Argentina) per animal respectively to induce ovarian superstimulation. Transrectal ultrasonography were performed on Days 2, 4, and 6; and ovum pick up (OPU) was performed on Day 6. Data was evaluated by one-way analysis of variance (ANOVA), repeated measures ANOVA, and 2-tailed Chi-square. The average size (mm) of follicles was greater (p≤ 0.05) in the 1000 IU group compared to the other groups. There was a greater (p≤ 0.05) number of follicles ≥ 7 mm in the 1000 IU group compared to the 500 IU group. Number of COCs collected on Day 6 and the COC recovery rate were not different among groups. In conclusion, a single dose of 1000 IU of eCG induced the best ovarian response resulting in larger and greater number of follicles at the time of OPU.

## Introduction

South American camelids, such as alpaca and llama, are economically important species adapted to *challenging high*-*altitude environments* in the Andes of South America (Cristofanelli et al., 2004). Despite that, deficiencies in traditional breeding schemes have contributed to lower genetic quality in these species (Kadwell et al., 2001; Frank et al., 2006). Reproductive technologies are tools that can be used to improve the genetics in llamas and alpacas (Miragaya et al., 2006). These techniques, that would allow the intensive use of selected breeders to increase in productivity of south American camelids, are still in development in these species. From these technologies, artificial insemination and embryo transfer are the most investigated technologies performed in South American camelids. The results have been variable, with a 3–67% fertility rate after AI in alpacas (Bravo et al, 2013) and 50–57% of conception rate in interspecies embryo transfer in both species (Sumar, 2013). Meanwhile *in vitro* embryo production (IVP) using oocytes collected from live animals, a highly efficient method of embryo production in other species, has not been successfully developed in South American camelids yet. 

According to the last report (Viana, 2022) of the International Embryo Technology Society (IETS), more embryos in the world are being produced using the in vitro technique. Some advantages of the IVP include the efficiency of producing higher number of embryos and pregnancies per unit of time, a wider range of potential female donors from which to retrieve oocytes, increased chances of obtaining the desired sex of offspring when sexed semen is used, and genetic improvement when genomic selection is used (Wheeler et al., 2006; Ferré et al., 2020). In South American camelids, reports of using IVP are limited. In these species, the main method reported for the obtention of cumulus-oocyte complexes (COCs) has been the dissection of ovaries from abattoir (Ratto et al., 2005; Landeo et al., 2017). The use of ultrasound-guided transvaginal follicle aspiration to perform the ovum pick up (OPU) to collect COCs was reported with limited success in llamas (Brogliatti et al., 2000, Berland et al., 2011). To date, there is not report of an offspring following transfer of IVP embryos produced from oocytes collected by OPU in South American Camelids. Failures occurring during ovarian superstimulation could be affecting the success of this technique in this species. Unfortunately, reports about ovarian superstimulation to perform OPU are scarce. Recently, researchers reported the use of 200 IU of equine chorionic gonadotropin (eCG) to superstimulate alpaca donors and collect oocytes through OPU (Landeo et al., 2022). The number of collected COCs was not high but the efficiency of in vitro embryo production was good (89%), therefore these results are encouraging in this species. 

Consequently, improving the protocols of superstimulation may have a positive effect on follicular development, hormonal secretion, and, ultimately, on oocyte quality. Therefore, the objective of this study was to evaluate the effect of superstimulatory protocols with three doses of eCG on ovarian response, follicular development and COCs collection in llamas.

## Methods

### Animals

Eighteen multiparous non-lactating adult (4–7 yo) female llamas with an average body condition score of 2.8 (range 1–5) and between 105 and 120 kg live weight were used for this study. The animals were housed in the farm of the laboratory of animal reproduction, Facultad de Medicina Veterinaria y Zootecnia, Universidad Nacional del Altiplano, Puno, Peru. From February to April 2021, all llamas were maintained in natural pastures (*Festuca sp*., *Agrostis tolucensis*, and *Stipa ichu*) supplemented with a daily portion of oat (cup of 100 gr) and hay (composed with alfalfa and ray grass) in the mornings, and water *ad libitum* to make sure that all their nutritional requirements are satisfied. Animals were handled according to protocols approved by the Animal Research Committee of Ethics, Universidad de Puno (Constance of authorization of ethics 104 committee N° CEBA-FMVZ-DARC-2020-19).

### Experimental design

**Figure 1:**
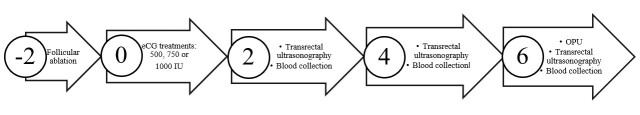
Study procedures sequence from follicular ablation to follicular aspiration.

Follicular ablation (FA) was performed in all experimental animals to induce the emergence of a new wave emergence. Briefly, animals were restrained in prone position and held with straps around the body on an examination table, and epidural anesthesia (2 mL of Lidocaine 2%, Medifarma, Lima, Peru) was given in the sacrococcygeal space. The vulvar region was thoroughly cleaned and a transvaginal probe (6.5 MHz, Sonostar SS8®, Guangzhou, China) with a needle guide was introduced into the vagina. Using a 19-G disposable OPU needle all follicles ≥ 5mm were aspirated (Ratto et al., 2003; Berland et al., 2011). Two days after FA (Day 0), animals were randomly allocated to three treatment groups (n = 6/group): i) 500 IU, ii) 750 IU, and iii) 1000 IU. The eCG (Novormon®, Syntex, Buenos Aires, Argentina) was given i.m. Transrectal ultrasonography was performed on Days 2, 4, and 6. Ovum pick up (OPU) was performed on Day 6 (Figure 1).

### Ovum pick up (OPU)

To perform the OPU in llamas, animals were restrained and prepared as described above for the FA. Transrectal ultrasonography was performed before aspirations to record the ovarian response and size of follicles of each animal. Subsequently, the vulvar region was disinfected, and the transvaginal probe (6.5 MHz, Sonostar SS8, Guangzhou, China) equipped with a needle guide was introduced into the fornix of the vagina. The ovaries were transrectally manipulated to bring them closer to the tip of the probe. Each follicle ≥ 3 mm of diameter was aspirated using a 19-G disposable OPU needle connected by silicon tubing to a 50 mL conical tube (BD Falcon, NJ, USA). Negative pressure of 100 to 120 mm Hg was applied using a vacuum aspiration pump. The collection medium consisted of Dulbecco's phosphate buffered saline (Biolife^TM^, Agtech, Inc. USA), supplemented with 0.2% bovine serum albumin,10000 IU/L heparin and 50 µg/L gentamicin. COCs were searched under stereoscope (Leica, leica Microsystems CMS Gmbh, Germany) at 40x magnification. The temperature of the COC searching room and medium was maintained at 25^0^ C. Recovered COCs were searched under stereomicroscope at 40X of zoom and maintained under holding media until further evaluation. 

### Statistical analysis

Number and size of follicles, and number of COCs were evaluated by one-way analysis of variance (ANOVA). Follicular development (Daily follicular growth) was evaluated by repeated measures ANOVA. The COC recovery rate (COC collected/follicles aspirated) was evaluated by 2-tailed Chi-square. The statistical analysis was performed using R 4.0.3 (R Core Team, 2020). Values are expressed as mean ± SEM, and P values of <0.05 were considered significant.

## Results

Daily follicular growth rate was not different among groups (p=0.092). However, the average size (mm) of follicles was greater (p≤ 0.05) in the 1000 IU group compared to the other groups (Table 1). There was no difference (p=0.061) in the number of follicles of 3-6 mm of diameter among groups on the Day of the OPU. Likewise, the number of COCs collected on Day 6 and the COC recovery rate were not different among groups. However, there was a greater (p≤ 0.05) number of follicles ≥ 7 mm in the 1000 IU group compared to the 500 IU group (Table 2). 

**Table 1:** Evaluation of follicular diameters during the mulitovulation process.

**Groups**	**Day 0**	**Day 2**	**Day 4**	**Day 6**
**500 IU**	1.84 ± 1.07	2.52 ± 0.18 a	4.07 ± 0.29 ^a^	6.13 ± 2.07 ^a^
**750 IU**	1.58 ± 0.62	2.66 ± 0.17 ^a^	5.15 ± 0.42 ^a,b^	6.99 ± 1.44 ^a^
**1000 IU**	1.87 ± 0.93	4.01 ± 0.21 b	5.83 ± 0.32 ^b^	8.08 ± 2.29 ^b^
*P-value*	0.061	0.043	0.033	0.0073

^a, b^ Different letters in rows show statistical difference.

**Table 2:** Evaluation of number of follicles, number of COCs collected and COC recovery rate in the different doses of eCG.

** **	**500 IU**	**750 IU**	**1000 IU**
**Number of follicles of 3-6 mm on the day of OPU**	4.4 ± 1.5	2.2 ± 1.3	3.0 ± 1.1
**Number of follicles ≥ 7mm on the day of OPU**	2.8 ± 1.9 ^a^	4.2 ± 1.8 a,b	7.4 ± 1.7 ^b^
**Number of COCs collected**	1.7 ± 0.2	2.00 ± 0.2	2.2 ± 0.6
**COC recovery rate**	42% (10/24)	50% (12/24)	48% (13/27)

^a, b^ Different letters in rows show statistical difference.

## Discussion

Performing OPU in non superstimulated female llamas may result in zero COCs collected. Therefore, ovarian superstimulation must be an important part of an OPU – IVP program to improve genetics in South American camelids. One hormone that has been used for ovarian superstimulation is the equine chorionic gonadotropin (eCG) which has both the FSH and LH effect (Murphy and Martinuk, 1991). Similar to FSH, eCG is given at the time of a new wave emergence to induce multiple follicular development in camelids (Huanca et al., 2009). Hormonal treatment with 1000 IU eCG to induce multiple follicular development for the purpose of superovulation and embryo collection has been developed in llamas (Aller et al., 2019; Zampini et al., 2020). However, a shorter protocol is needed for the purpose of in vitro embryo production. Recently, a superstimulatory protocol with 200 IU of eCG for OPU and IVP was developed in alpacas (Landeo et al., 2022). The use of that protocol resulted in 60 follicles ≥5 mm aspirated from 13 alpacas. Therefore, in the present study, we wanted to compare different eCG dosages to induce multiple follicular development in llamas. Interestingly, we found that eCG has a superstimulatory effect in a dose-dependent manner in llamas. 

In this work, a single dose of 1000 IU of eCG was efficient to produce an average of 7.4 follicles (≥ 7mm) on the day of the OPU in llamas. Which was the greatest number of follicles in this study. In an early study, it was found that a single injection of 1200 IU of eCG produced an average of 27 follicles (≥6 mm) in alpacas (Ratto et al., 2007). Probably, the higher dose of eCG in the latter may have caused a greater superstimulatory effect compared to our study. However, the differences may be attributed to the different minimum size of follicles reported in the two studies or the source of the eCG. On the other hand, the average size of the follicles on the day of OPU was the highest in the 1000 IU group. This is important since it was found that the size of the follicle is positively correlated to oocyte quality (Blondin et al., 2012). Therefore, aspirating follicles that are in the dominance state or greater, it may help to improve IVP results in llamas and alpacas. Future studies are needed to evaluate this statement. 

Interestingly, recovery rate was not different among groups in this study despite the larger number of follicles found in the 1000 IU group. In cattle, the OPU skill of the practitioner is one important factor that has been shown to affect the success of the recovery rate (Bols and Stout, 2018). In our study, the technician performing the OPUs encountered difficulties during the procedures, which may be related with the small size of the rectum of the llamas. During the OPU procedures, there is also a possibility that some of the largest follicles may have invertedly ruptured due to the manipulation of the ovaries and therefore affecting the recovery rate. Further studies need to be done to improve the technical difficulties of the OPU procedure in this species.

In conclusion, a single dose of 1000 IU of eCG induced the best ovarian response resulting in greater number and larger follicles at the time of OPU in llamas. These data suggest that there may be a dose-dependent superstimulatory effect of the eCG in llamas. Therefore, a higher dose of eCG may result in a greater ovarian response in this species. Nevertheless, further studies are needed to improve the number of COCs collected from superstimulated llamas.
